# Broadband PM6Y6 coreshell hybrid composites for photocurrent improvement and light trapping

**DOI:** 10.1038/s41598-024-63133-5

**Published:** 2024-06-12

**Authors:** S. Sanad, AbdelRahman M. Ghanim, Nasr Gad, M. El-Aasser, Ashraf Yahia, Mohamed A. Swillam

**Affiliations:** 1 Department of Physics, Faculty of Science, Ain Shams University, Cairo, 11566 Egypt; 2 Department of Physics, School of Sciences and Engineering, The American University in Cairo, New Cairo, 11835 Egypt

**Keywords:** Organic solar cell, PM6Y6, LSPR, Refractory metals, Absorption spectra, TiN, Materials science, Optics and photonics

## Abstract

Our research focuses on enhancing the broadband absorption capability of organic solar cells (OSCs) by integrating plasmonic nanostructures made of Titanium nitride (TiN). Traditional OSCs face limitations in absorption efficiency due to their thickness, but incorporating plasmonic nanostructures can extend the path length of light within the active material, thereby improving optical efficiency. In our study, we explore the use of refractory plasmonics, a novel type of nanostructure, with TiN as an example of a refractory metal. TiN offers high-quality localized surface plasmon resonance in the visible spectrum and is cost-effective, readily available, and compatible with CMOS technology. We conducted detailed numerical simulations to optimize the design of nanostructured OSCs, considering various shapes and sizes of nanoparticles within the active layer (PM6Y6). Our investigation focused on different TiN plasmonic nanostructures such as nanospheres, nanocubes, and nanocylinders, analyzing their absorption spectra in a polymer environment. We assessed the impact of their incorporation on the absorbed power and short-circuit current (Jsc) of the organic solar cell.

## Introduction

OSCs play a vital role in the development of low-cost, high-efficiency thin-film solar cells by enabling the production of solar cells with comparable efficiency to their inorganic counterparts^1,2^. The efficiency of solar cells is influenced by multiple parameters. Light absorption, carrier separation, carrier generation, carrier diffusion, and eventually carrier collection are among these parameters. Thus, improving light absorption efficiency is the major part of enhancing total power conversion efficiency (PCE)^3–6^. The limited light absorption efficiency in OSCs may be attributed to the short carrier diffusion length and low exciton mobility^7^.

The use of small molecules and solution-processed bulk-heterojunction (BHJ) polymer/fullerene blends has been investigated for the production of OSCs^8^. The active layer of a BHJ comprises a combination of donor and acceptor materials^9^. These structures were employed to address the issue of limited exciton lifetime, with the aim of enhancing charge generation and separation in the BHJ system^10,11^. Nonfullerene acceptors (NFAs) with an acceptor–donor–acceptor (A–D–A) structure, which consists of an electron-donating fused-ring core as the D unit and electron-drawing end groups as the A unit, have been extensively investigated and exhibit outstanding photovoltaic performance ^12,13^.

PM6, also known as PBDB-TF or PBDB-T2F, is a copolymer of the D–π–A type. It has an optical bandgap of ≈ 1.80 eV, a strong absorption spectrum in the range of 500 nm to 700 nm, and the highest occupied molecular orbital (HOMO) energy of ≈ 5.50 eV. The D, π, and A units in this copolymer are represented by benzodithiophene (BDT), thiophene, and benzodithiophene-4,8-dione (BDD), respectively^14^. The star polymer PM6 has been shown to exhibit desirable donor properties, making it suitable for blending with various non-fullerene acceptors (NFAs), including both small molecules and polymers^15^. In the year 2019, researchers successfully synthesized a unique NFA Y6 compound with an A–DA'D–A molecular structure^12,16^. This design had a center-fused ring with an electron-deficient core. The incorporation of the electron-deficient group onto the central π-conjugated structure results in a strong absorption at near-infrared, with an absorption onset located at 931 nm^17^. Additionally, Y6 exhibits the highest occupied molecular orbital (HOMO) energy level of − 5.65 eV and the lowest unoccupied molecular orbital (LUMO) energy level of − 4.10 eV. The Y6-based OSCs demonstrated a significant PCE of 15.7% when combined with the commonly used polymer donor PM6^17,18^. PM6:Y6 polymer material has become essential for OSCs to obtain high efficiency from solar energy. Due to the low carrier mobility and short exciton diffusion length of organic materials, the BHJ based on the PM6:Y6 active layer with a limited thickness of 100 nm achieves a PCE greater than 18%^12,19^.

The absorption efficiency can be enhanced by the use of several light trapping methods, including surface texturing^20^, photonic crystals^21^, and plasmonic nanostructures^2,22–24^. Plasmonic solar cells have considerable potential as configurations capable of addressing the problem of low absorption in thin films by using metallic nanostructures. The nanostructures possess the ability to concentrate light inside the active layer, leading to an increased optical path length and therefore higher absorption efficiency. Most of the light trapping approaches reported for OSCs utilize metal plasmonic nanostructures^25,26^. This enhancement can result in making OSCs more competitive with traditional silicon-based solar cells. OSCs are known for their potential low-cost manufacturing processes compared to inorganic solar cells. PM6Y6 polymer with nanoparticles offers flexibility and versatility in terms of form factor and application. This characteristic makes the OSC suitable for a wide range of applications, including wearable electronics, building-integrated photovoltaics, and portable devices, thus satisfying diverse industry and community needs. OSCs are considered more environmentally friendly than traditional solar cells due to their lower energy consumption and carbon footprint during production.

It’s found that incorporating plasmonic nanoparticles into organic solar cells (OSCs) can be beneficial^2,25,27^. This is achieved through near-field enhancement and scattering effects caused by localized surface plasmon resonance (LSPR). By altering the size, materials, and shapes of the nanoparticles, the light absorption can be increased, leading to improved PCE as the nanoparticles aid in the separation of excitons and collection of charge carriers. However, the plasmonic effect is limited by the narrow LSPR band of individual nanoparticles. Therefore, it is advantageous to design a range of the solar spectrum from visible to NIR regions in order to achieve a collective plasmonic effect, resulting in high-performance OSCs^6,25,28,29^.

This study highlights a novel category of materials that have the potential to enhance the absorption of a wide range of wavelengths in PM6:Y6. These materials overcome the drawbacks associated with metals, such as losses due to interband transitions, high costs, incompatibility with C-MOS technology, and chemical instability^30–32^. In recent times, there has been a growing interest in refractory plasmonics owing to its ability to stimulate surface plasmon polaritons (SPPs) and LSPs^28,33^.

The conductive ceramics known as nitrides of group IVb-Vb-VIb transition metals (such as TiN, ZrN, HfN, VN, NbN, TaN, MoN, and WN) have unique properties including high electronic conductivity, extremely high melting points, and a wide range of work function values^34^. These materials have been considered for various electronic applications since the early 1980s, including use as diffusion barriers in integrated circuit metallizations, Ohmic contacts on compound semiconductors, and thin film resistors. Recently, TiN has emerged as a promising candidates for plasmonic applications. However, the potential plasmonic activity of other transition metal nitrides (TMN) remains an important question that requires further investigation^2,34^. This is primarily attributed to its possession of a high-quality factor for localized surface plasmon resonance (LSPR), which is similar to that of well-established plasmonic metals such as silver (Ag) and gold (Au)^33^. Furthermore, it has been shown that transition metal nitrides exhibit compatibility with C-MOS fabrication techniques, as evidenced by previous studies^35^. The use of C-MOS technology for the fabrication of nanoparticles offers advantages such as precise control over size and shape, low cost, the ability to scale up for mass production, integration with existing semiconductor processes, and the potential for creating novel electronic and optoelectronic devices.

## Methodology and design

**Figure 1:**
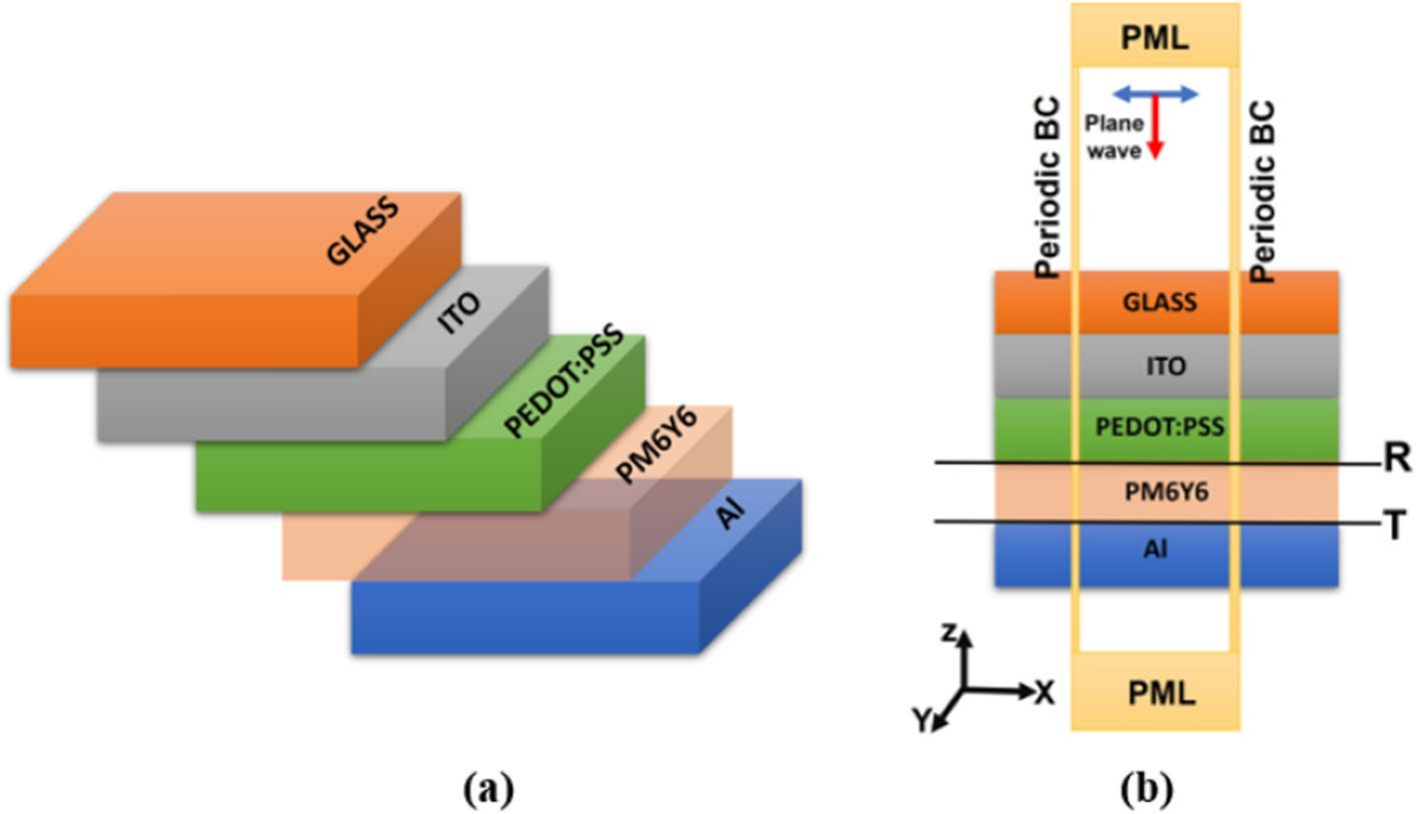
(**a**) 3D structure of the proposed OSC. (**b**) Computational domain with the boundary conditions of OSC.

The proposed planar OSC design shown in Fig. 1a consists of five layers, each serving a specific purpose. The front electrode is made of indium tin oxide (ITO) and has a thickness of 180 nm. The active layer, which is responsible for absorbing light and generating electricity, is composed of a blend of PM6 and Y6 with a thickness of 250 nm and a ratio of 1:1.2. Acting as a hole transport layer (HTL), PEDOT: PSS is applied with a thickness of 50 nm. The back electrode is made of aluminum (Al) and has a thickness of 180 nm. Finally, the entire structure is supported by a glass substrate with a thickness of 200 nm. This design (ITO/PEDOT: PSS/PM6:Y6/Al) aims to optimize the performance and efficiency of the organic solar cell.

Numerical simulations were conducted using the commercially available Lumerical finite difference time domain (FDTD) solutions software^29,36^. Figure 1b illustrates the schematic computational domain for the organic solar cell structure. To prevent unwanted reflections in the stack direction, perfectly matched layer (PML) boundary conditions were applied to the glass substrate and aluminum (Al) layers. Periodic boundary conditions were set along the *x* and *y* directions, as shown in Fig. 1b. In order to minimize computation time, the 3D simulation box is bounded by anti-symmetric, symmetric boundary conditions. In order to achieve fine resolution, mesh override sections covering distinct volumes of the structure were employed. The incident light was modeled as a plane wave propagating normally along the *z* direction, with a wavelength ranging from 300 to 1100 nm. The solar cell utilized transparent conducting materials, with ITO serving as the anode and Al as the cathode. A buffer layer of poly(3,4-ethylenedioxythiophene): poly(styrenesulfonate) (PEDOT: PSS) and an active layer of PM6Y6 in a 1:1.2 weight ratio were employed. The refractive indices of glass, aluminum, PEDOT:PSS, ITO, and PM6Y6 were obtained from the literature^37,38^. In order to estimate the transmittance (*T*) and reflectance (*R*) spectra, two FDTD power monitors are utilized as shown in Fig. 1b. The computation of the power absorbed (*P*_*abs*_) involves utilizing the transmittance and reflectance spectra as:

1 $$ P_{abs} = { 1} - R - T $$

to compute the quantum efficiency (QE(λ)) of the proposed solar cell, the following equation can be used^2^:

2 $$QE(\lambda ) = \frac{{P}_{abs}\left(\lambda \right)}{{P}_{in}(\lambda )}$$

where ${P}_{in}(\lambda )$ is the power of light incident. When employing the FDTD method, the light source operates as the excitation. The light source is positioned directly above the material, and the numerical wave generated by the light source propagates towards the structure. During this propagation, a portion of the wave is transmitted through the material while another portion is reflected. FDTD is capable of calculating the generation rate of light^39^, and its mathematical expression can be given by equation^40,41^

3 $$G\left(\overrightarrow{r}\right)= \frac{{P}_{abs}\left(\overrightarrow{r}, \omega \right)}{\hslash \omega }=\frac{{-0.5|E\left(\overrightarrow{r},\omega \right)|}^{2}Im[\varepsilon \left(\overrightarrow{r},\omega \right)]}{\hslash }$$

where *ω* is the angular frequency; *E* represents the electric field strength; *ε* is the dielectric constant; ℏω represents the energy of a photon. Additionally, *P*_*abs*_ denotes the spatial power density of absorption and it can be defined as^41^. The generation rate is a crucial parameter in solar cell operation as it determines the number of electrons generated at each point in the device due to the absorption of photons.

4 $${P}_{abs}\left(\overrightarrow{r}, \omega \right)= {-0.5|E\left(\overrightarrow{r},\omega \right)|}^{2}Im[\varepsilon \left(\overrightarrow{r},\omega \right)]$$

Given that every electron–hole pair produced within the active layer plays a role in generating photocurrent, the short-circuit current density (*J*_*SC*_) of a solar cell can be estimated by^42^

5 $${J}_{sc}= \frac{e}{hc}\int A\left(\lambda \right)AM1.5(\lambda )d\lambda $$

where* e* represents the electronic charge, *h* denotes the Planck constant, c is the speed of light in a vacuum, and AM1.5G refers to the solar spectrum.

## Results and discussions

In order to validate the simulation results calculated by the 3-D FDTD method, a thin film Si solar cell designed by Hanif Kazerooni, et al.^43^. is initially considered. The unit cell of the studied solar cell structure, including a back structure composed of silver (Ag), a silicon (Si) layer with a thickness of 200 nm, and a conventional antireflection coating made of silicon nitride (Si_3_N_4_) with a thickness of 80 nm. On the Ag back contact of the structure, square silver nanoridges with a height of 50 nm are positioned, forming a Sierpinski pattern of the second order.

**Figure 2:**
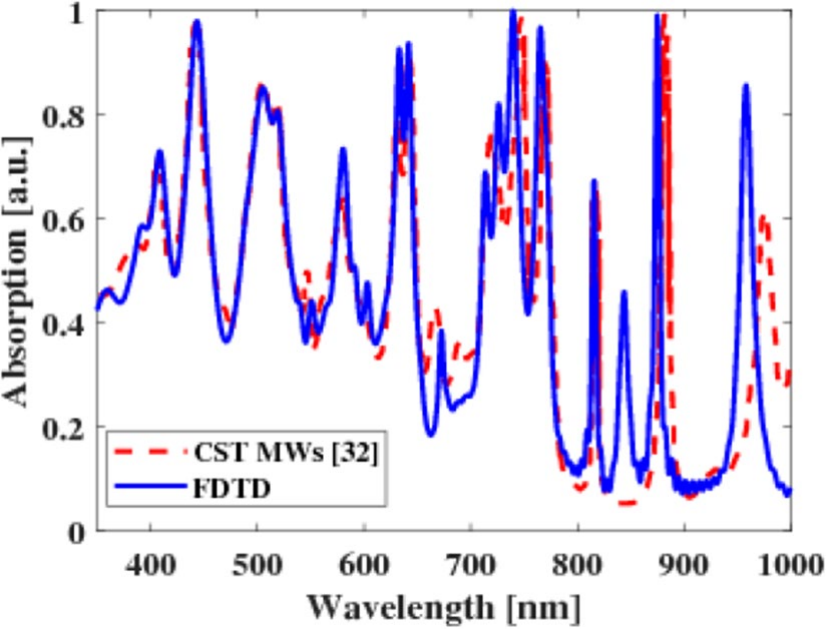
The absorption spectra as a function of the wavelength of the thin film Si solar cell^43^ and by FDTD simulation.

The dielectric function of silver (Ag) in this study is assumed to follow the Drude model, with a value of ${\varepsilon }_{\infty }$ = 3.7. The plasma frequency (*ω*_*p*_) of Ag is calculated to be 1.38 × 10^16^ rad/s, and the collision frequency (*γ*) is measured at 2.73 × 10^13^ 1/s. The refractive index of silicon (Si_3_N_4_) is considered to be *n* = 2, while the refractive index of Si is obtained from Palik's data^37,43^. The wavelength-dependent absorption spectra computed by the FDTD approach and by Hanif Kazerooni et al. are shown in Fig. 2. It can be noted that a good agreement can be observed between our results and those published, indicating that our model is accurate.

In order to evaluate the performance of organic solar cells, a comparison was made first between two polymers (active layer): P3HT:PCBM and PM6Y6. These polymers are commonly used in the fabrication of organic solar cells due to their unique properties. P3HT:PCBM is a blend of poly(3-hexylthiophene) (P3HT) and [6,6]-phenyl-C61-butyric acid methyl ester (PCBM), while PM6Y6 is a polymer with a different chemical structure. The geometrical parameters of the proposed design of the OSC are as follows: a 200 nm thick protective layer of SiO_2_ glass is utilized, followed by a front electrode made of Indium Tin Oxide (ITO) with a thickness of 180 nm. The reason for selecting the ITO as a transparent conducting oxide is due to its remarkable electrical conductivity and optical transparency^44^. It has an optical band gap of around 3.6 eV and a plasma frequency in the near-infrared spectral range. ITO has proven to be an advanced semiconductor material that has demonstrated its potential in a wide range of electrical and optical applications. While ITO reflects infrared light to a certain extent, it exhibits exceptional transparency in the visible spectrum due to its unique characteristics and notable features^44,45^. The subsequent layer is PEDOT:PSS, serving as a hole transport layer (HTL) and measuring 50 nm in thickness. Next is the active layer, which has a thickness of 250 nm. Finally, there is an anode layer composed of Aluminum (Al) with a thickness of 120 nm as shown in Fig. 1.

**Figure 3:**
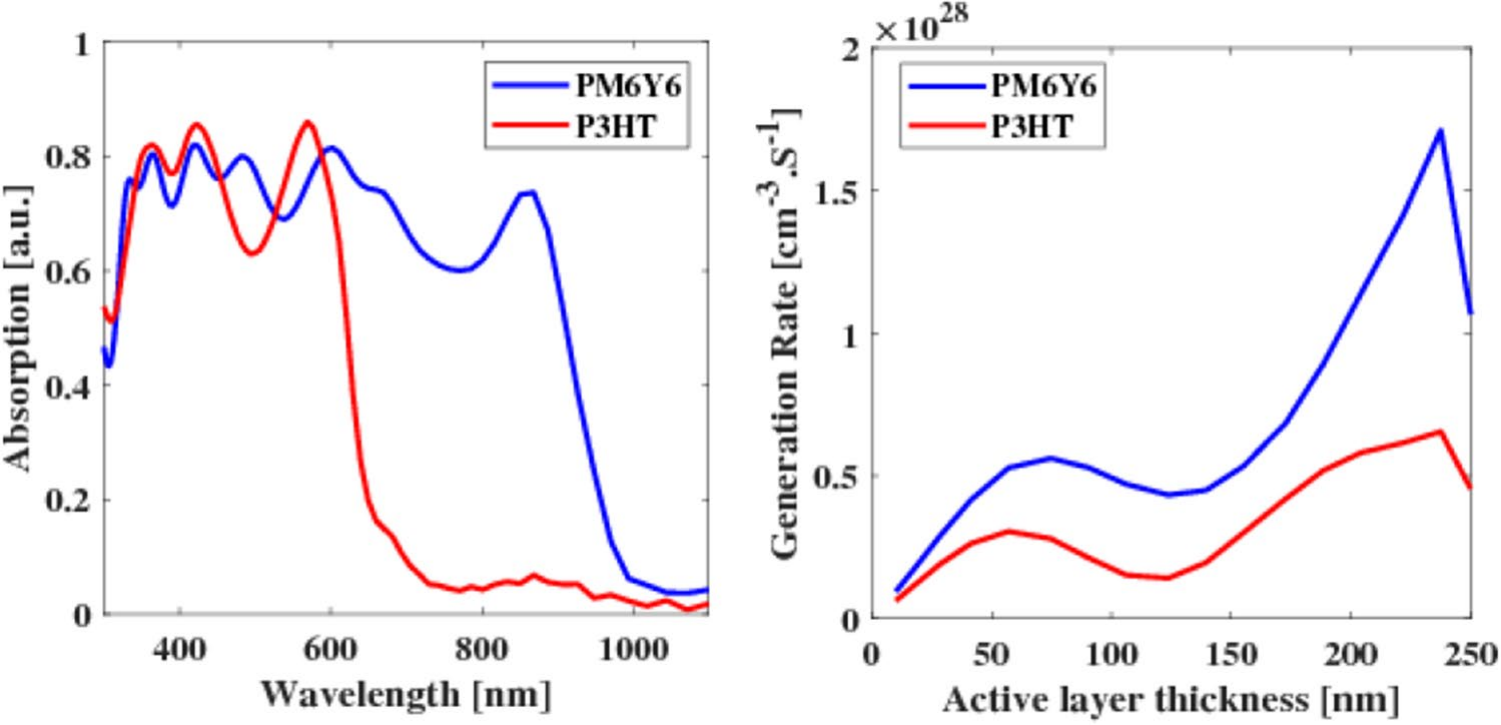
Comparison between PM6Y6 and P3HT in the case of (**a**) absorption spectra as a function of wavelength and (**b**) generation rate as a function of the layer thickness.

In this study, the power absorbed in the *z*-direction is calculated using the FDTD method through Lumerical software packages. The OSC design under investigation is illuminated by a plane source that oscillates along the *z*-direction. Figure 3 presents a comparison of the absorption characteristics between OSCs incorporating PM6Y6 and P3HT as the active layer. It can be observed that PM6Y6 demonstrates a broad absorption range from 300 to 1100 nm, in contrast to P3HT. Figure 3a shows that P3HT exhibits absorption levels exceeding 0.8 between 300 and 570 nm, with absorption decreasing beyond 570 nm. In comparison, PM6Y6 material maintains an absorbed power of approximately 0.8 from 300 to 600 nm, decreasing to about 0.7 from 600 to 870 nm. This implies that PM6Y6 has the ability to absorb a wider spectrum of light in the visible range compared to P3HT as shown in Fig. 3a. The expanded absorption range of PM6Y6 indicates its potential to capture a larger portion of the solar spectrum, which can be beneficial for improving the overall efficiency of OSCs. This enhancement is attributed to the combination of the PM6 and Y6 in a single layer, which leads to a significant increase in the electrostatic interface field for Y6. This compensates for the coulomb dissociation barrier, resulting in a high *J*_*sh*_ (short-circuit current density)^12^. Consequently, barrier-free dissociation occurs, leading to a narrow distribution of electronic trap states compared to other studied organic blend systems. Additionally, the presence of intra-band gap states with low energetic disorder contributes to reduced energetic losses, including charge carrier trapping into any state. This enhances the performance of the device, ensuring uniformity and rigidity, promoting efficient charge generation, minimizing voltage losses in this low energy offset system, and reducing charge recombination to Y6^14,16^. PM6 has a large bandgap of 1.80 eV and an intense absorption spectrum between 500 and 700 nm. Y6 consists of an electron-deficient central core composed of dithienothiophen[3,2-b]-pyrrolobenzothiadiazole and flanking 2-(5,6-Difluoro-3-oxo-2,3-dihydro-1H-in den-1-ethylidene) malononitrile units^12,46^. The embedding of the electron-deficient group on the central π-conjugated core, forming an A–DA'D–A configuration, gives Y6 a strong near-infrared light absorption with an absorption onset at 931 nm, corresponding to a narrow optical bandgap of 1.33 eV, and a strong absorption coefficient of 1.07 × 10^5^ cm^47^. High photocurrent responses in OSC devices are contingent upon the strong and well-complementary light absorption spectra of PM6 and Y6.

In the case of PM6Y6 and P3HT, simulations have been conducted to analyze the position-dependent generation rate within the active layer. These simulations provide valuable insights into the performance and efficiency of the solar cells made from these materials. In the active layer of an organic solar cell, it is anticipated that a higher generation rate $G\left(\overrightarrow{r}\right)$ will lead to an increase in the short-circuit current (*J*_*sc*_)^41^. Consequently, the active layer thickness that results in the widest and brightest spot for $G\left(\overrightarrow{r}\right)$ can be considered as having the optimal performance. This implies that maximizing the generation rate within the active layer is crucial for achieving higher *J*_*sc*_ values. By selecting an active layer thickness that allows for the broadest and most intense spot of $G\left(\overrightarrow{r}\right)$, the organic solar cell can operate at its highest efficiency and deliver enhanced power output. The organic layer PM6Y6 exhibits a significantly higher generation rate in comparison to P3HT as can be seen in Fig. 3b. This characteristic enables PM6Y6 to create a broader and more intense spot within the organic solar cell. As a result, the solar cell can operate at its peak efficiency, leading to an enhanced power output. The higher generation rate of PM6Y6 contributes to a more efficient conversion of sunlight into electrical energy, making it a promising candidate for improving the performance of organic solar cells.

**Figure 4:**
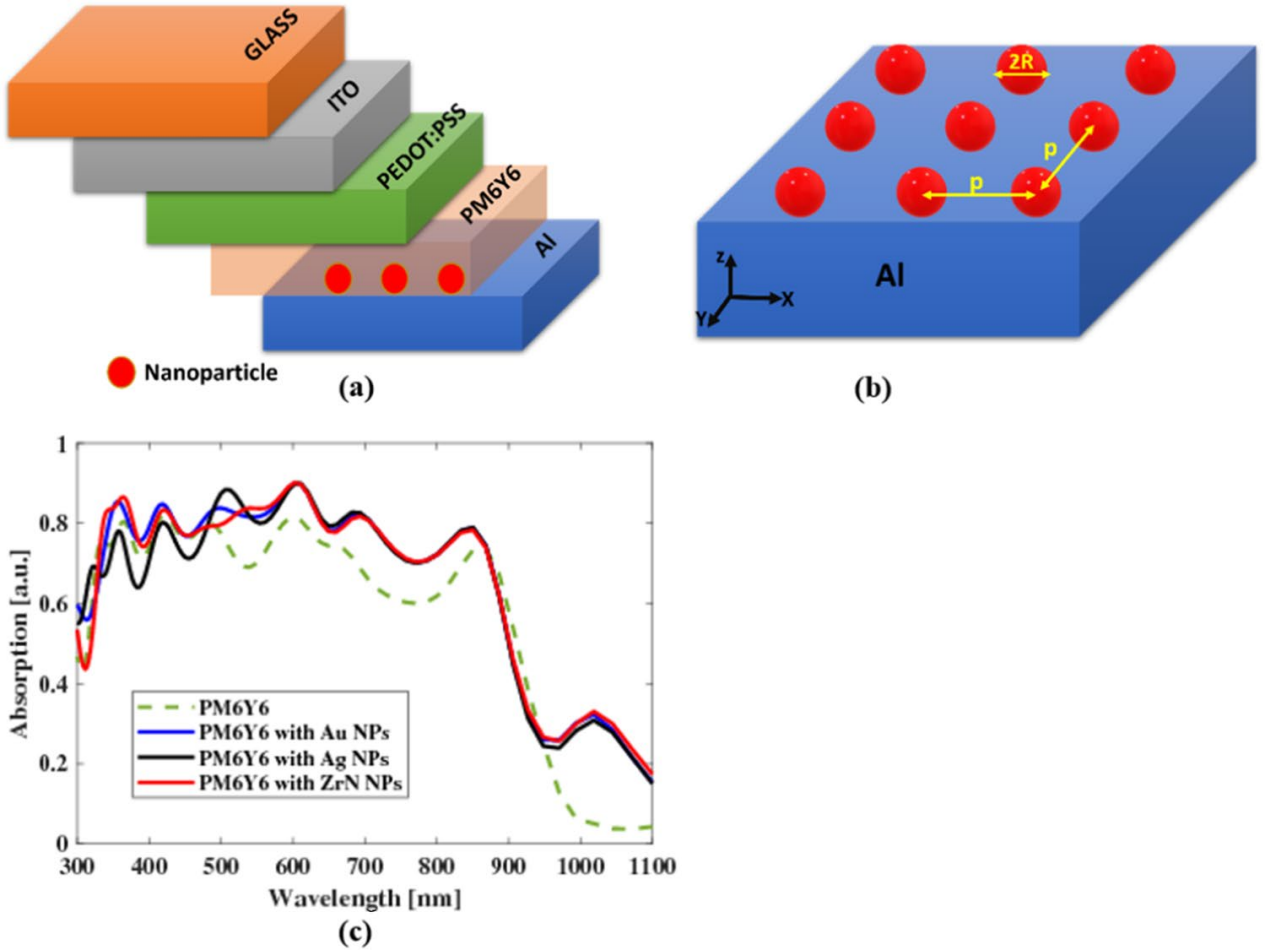
(**a**) 3D planar structure of the proposed OSC with nanosphere NPs, (**b**) the schematic diagram of the square array of nanospheres with the lattice constant labeled as *p* and each nanosphere has a radius of *R*, and (**c**) Absorption spectra of PM6:Y6 without NPs and PM6:Y6 with Au NPs, Ag NPs, and ZrN NPs.

In this paper, a square periodic array of plasmonic nanoparticles (NPs) with various sizes and shapes was embedded in the active layer. These NPs were strategically placed at different locations, including the active layer and the buffer layer, to create LSPR. As a result, the NPs functioned as scattering centers for the incident light, causing the light to scatter at different angles. This scattering effect elongated the optical paths, leading to the trapping of more light and consequently increasing the optical absorption. By utilizing this approach, the overall efficiency of the system can be significantly improved. In our study, we investigated different configurations of OSCs that utilized PM6Y6 as the active material. The initial planar structure, as shown in Fig. 1a, was modified by incorporating NPs into the active layer. The other configurations were obtained by embedding a square periodic array of plasmonic NPs with varying shapes and sizes in the active layer. Figure 4a depicts the OSC with nanospheres embedded into the polymer layer. By exploring these different configurations, we aimed to evaluate the impact of NPs on the performance and efficiency of the OSCs.

In the present work, our focus is on comparing the performance of OSCs utilizing the bulk heterojunction system of PM6Y6. Specifically, we aim to investigate the differences in performance when the active layer of the OSC without nanoparticles, as compared to when it incorporates spherical metal NPs such as gold and silver. These metal NPs have a lattice constant of *p* = 70 nm and a radius of *R* = 25 nm as shown in Fig. 4b. The choice of gold and silver NPs is based on their unique properties, including their plasmonic characteristics and high electrical conductivity. These properties make them attractive candidates for improving light absorption and charge transport within the OSCs. Recently, there has been an increased focus on refractory plasmonics due to their ability to stimulate surface plasmon polaritons (SPPs) and LSPRs^33^. Among these refractory plasmonics, zirconium nitride (ZrN) has emerged as a promising option for LSP applications. This is primarily attributed to its high-quality factor for LSPR, which is comparable to plasmonic metals like silver (Ag) and gold (Au)^2,33^. Furthermore, it is worth noting that transition metal nitrides, including ZrN, are known to be compatible with C-MOS fabrication processes^48^.

Figure 4c illustrates the absorption spectra of OSCs that incorporate the plasmonic nanostructures with a radius of *R* = 25 nm. The absorption spectra are plotted as a function of wavelength. Additionally, for comparison purposes, the absorption spectrum of a planar OSC, without any plasmonic nanostructure, is also depicted in Fig. 4c. The results of the study indicate that the incorporation of a plasmonic nanostructures array leads to a significant enhancement in light absorption within the active layer of the OSCs. The absorbed power reaches up to 0.9 between 300 and 600 nm for both PM6Y6 without NPs and PM6Y6 with added NPs. However, at a wavelength of 600 nm, the absorbed power for PM6Y6 decreases to 0.8, while the polymer with NPs maintains an absorption level of around 0.9 (with an enhancement of 10%). Moving to a wavelength of 1000 nm, the absorbed power of the polymer containing metal NPs (Au and Ag) increases to about 0.3, whereas with ZrN, it reaches approximately 0.33. These results indicate that the presence of plasmonic nanostructures enhances absorbed power in the near-infrared (NIR) range. This improvement in light absorption is observed across a broad range of wavelengths, indicating that the plasmonic nanostructures effectively enhance the light-harvesting capabilities of the OSCs. The simulated results revealed that the OSC incorporating ZrN NPs embedded within the active layer exhibited the highest absorption spectra. This indicates that the presence of ZrN NPs significantly enhances the light absorption capabilities of the OSC and efficient interaction with incident light.

In this paper, The unique combination of electronic properties, stability, and refractory nature of TiN has led to its utilization in OSC applications. These include Schottky contacts, superconducting devices, and optoelectronic devices^34^. One exceptional advantage is its compatibility with CMOS technology, thanks to high electron mobility and refractory characteristics. This compatibility allows for easy integration and scaling of TiN in realistic electronic devices. In particular, TiN has the potential to enhance the integration of plasmonics into CMOS technology for optical communications by replacing incompatible materials such as Au and Ag. TiN stands out among the various refractory plasmonic materials due to its potential as a possible option for LSP applications. One of its key advantages is its high-quality factor for LSPR, which is comparable to that of widely used plasmonic metals. This characteristic makes TiN a promising candidate for controlling LSP effects in various applications.

**Figure 5:**
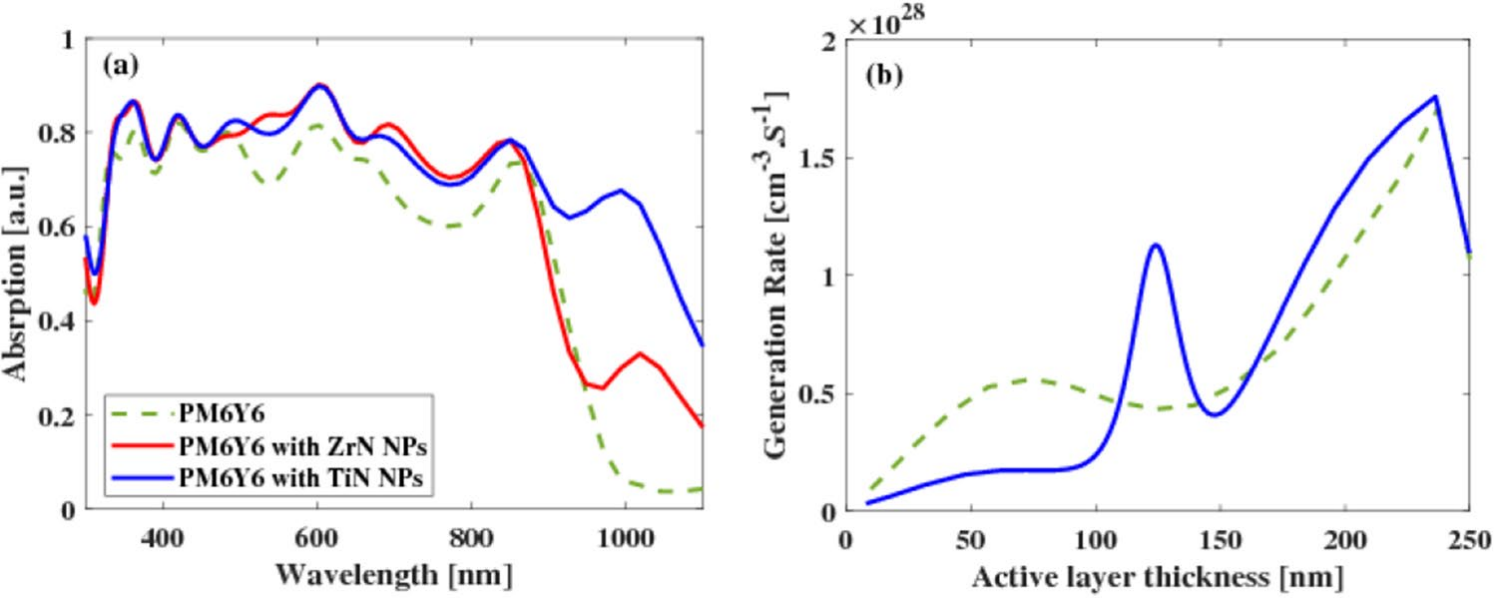
(**a**) Wavelength dependence absorption spectra of PM6Y6 with ZrN NPs compared with PM6Y6 with TiN NPs and (**b**) generation rate as a function of the layer thickness for PM6Y6 without NPs and PM6Y6 with TiN NPs.

In Fig. 5a, the optical absorption spectra of the photoactive layer consisting of PM6:Y6 with ZrN nanoparticles are depicted, along with the absorption spectra for PM6:Y6 with TiN nanoparticles. Notably, TiN nanoparticles exhibited a distinct LSPR peak at approximately 600 nm and another peak at 1000 nm. In the context of this study, the incorporation of TiN nanoparticles within the PM6:Y6 layer was employed to achieve a significant enhancement in absorption across a broad spectrum both in the visible and NIR regions, in organic solar cells (OSCs). This utilization of TiN nanoparticles in the PM6:Y6 layer resulted in a broadband improvement of light absorption, thereby enhancing the overall performance of OSCs. For PM6Y6 with NPs (ZrN and TiN), the absorbed power reaches 0.9 between 300 and 600 nm. However, the absorption levels differ as the wavelength increases. Specifically, for PM6Y6 with ZrN NPs, absorption decreases from 0.45 to 0.33 between 900 and 1000 nm. In contrast, PM6Y6 with TiN NPs maintains a higher absorption level of 0.65 (with an enhancement of 32%) in this range, decreasing to 0.35 at 1100 nm, which is higher than the absorption observed with ZrN NPs at around 0.17.

Figure 5b presents the generation rate of the PM6Y6 in comparison to the PM6Y6 with the inclusion of TiN NPs. The figure clearly demonstrates that the introduction of TiN NPs into the active layer significantly enhances the generation of electrons, thereby improving the overall performance of the OSCs. This observation highlights the positive impact of incorporating TiN nanoparticles on the efficiency and functionality of OSCs.

**Figure 6:**
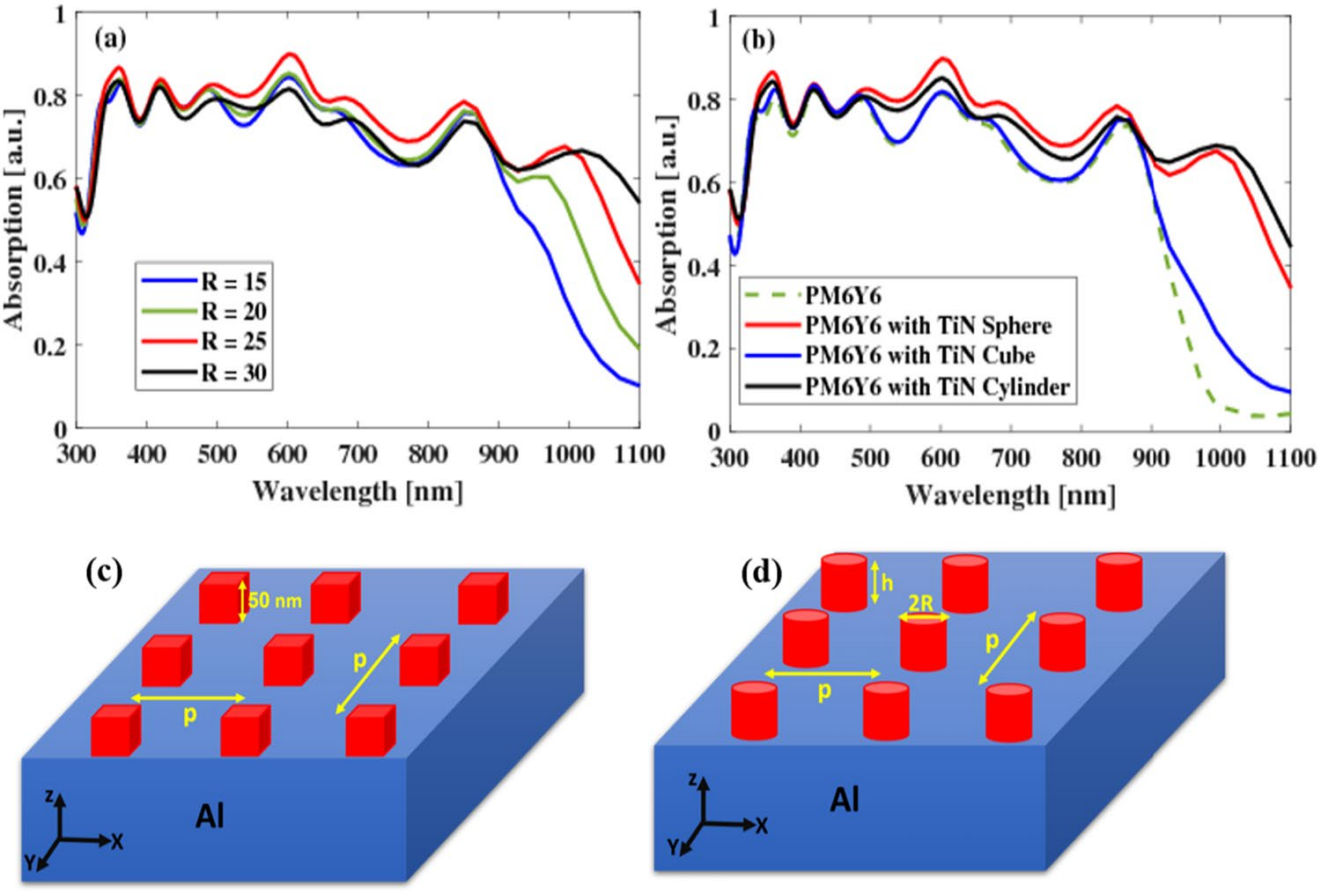
Absorption of PM6Y6 with TiN NPs for (**a**) different radii for nanospheres, and (**b**) different geometries. (**c**, **d**) are schematic views of the square periodic array of nanocubes and nanocylinders, respectively.

One of the key advantages of the proposed OSC design is the flexibility to adjust the design parameters, specifically the radii of the TiN nanospheres, to achieve high absorption spectra. In order to investigate the impact of nanosphere radius on the absorption characteristics, the suggested design was examined. Figure 6a illustrates the absorption spectra of the OSC design for various radii. It is observed that by tuning the radii of the TiN nanospheres, the absorptance of the OSC is significantly enhanced, reaching its maximum value at a radius of approximately 25 nm. In this study, we investigate three different configurations of OSCs utilizing PM6Y6 as the active material. These configurations involve the incorporation of TiN NPs in the form of square periodic arrays with varying shapes within the active layer as shown in Fig. 6b. Specifically, we consider nanospheres with a radius of *R* = 25 nm, nanocubes with dimensions of 50 × 50 × 50 nm^3^ (see Fig. 6c), and nanocylinders with a radius of *R* = 25 nm and a height of approximately *h* = 33 nm (see Fig. 6d). Notably, all shapes exhibit an enhancement in their absorption curves compared to the base structure without NPs. Additionally, certain shapes demonstrate a higher degree of optical absorption than others. Among all the shapes considered, the nanosphere NPs exhibit the highest absorption spectrum, better than both the base structure and the other NP shapes as depicted in (Fig. 6b).

**Figure 7:**
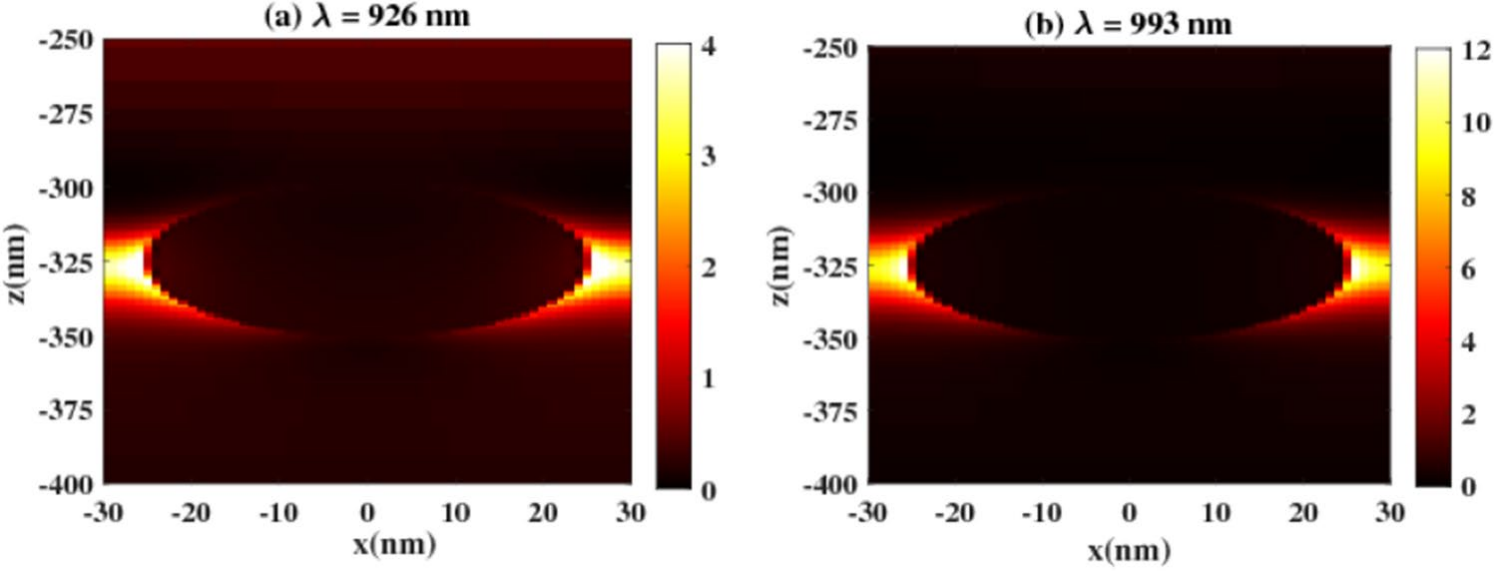
Electric field profile inside the structure with TiN nanosphere at (**a**) 926 nm and (**b**) 993 nm.

The absorption spectra shown in Fig. 6b, can be deduced through an analysis of the electric field distribution at different wavelengths. Specifically, a light localization is observed around the nanosphere at a wavelength of 926 nm; indicating the ability of TiN to induce a strong LSPR, as depicted in Fig. 7a. On the other hand, at a wavelength of 993 nm, a significantly higher field is observed around the NP, as shown in Fig. 7b. This observation explains the significant increase in the absorption spectra observed at the NIR region.

**Figure 8:**
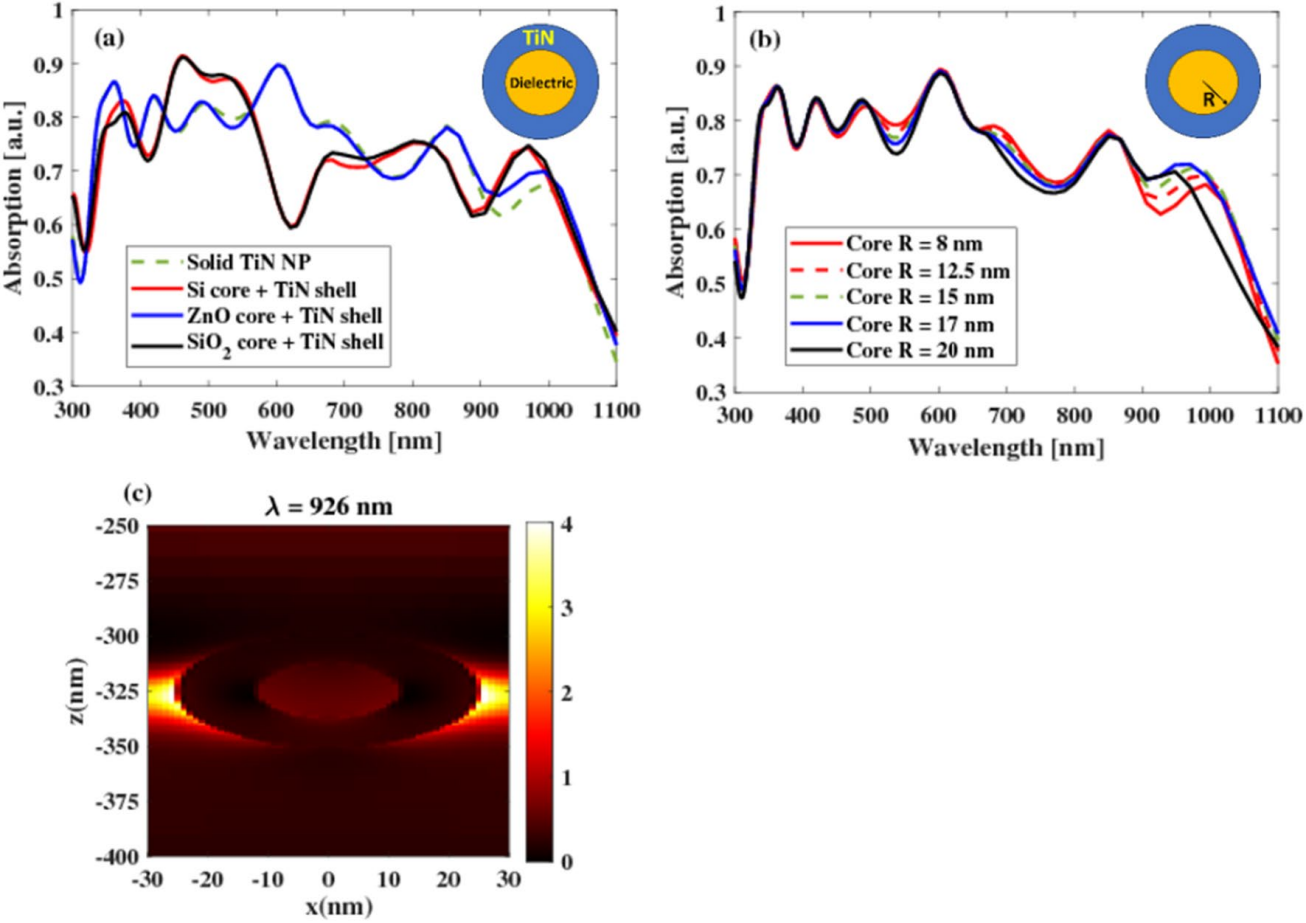
(**a**) Optical absorption of PM6Y6 with solid TiN NPs, PM6Y6 with TiN–Si core–shell, PM6Y6 with TiN–ZnO core–shell, and PM6Y6 with TiN–SiO_2_ core–shell while (**b**) is the absorption with varying the core radius and (**c**) the electric field for the PM6Y6 with TiN–ZnO core–shell when the core radius is 17 nm at a wavelength of 926 nm.

Metallic nanoshells have been extensively investigated and employed in various types of solar cells, including dye-sensitized solar cells^49^, Si solar cells^50^, and perovskite solar cells^51^. These nanoshells have shown promising potential in enhancing the performance of solar energy conversion devices. In this study, we introduce a novel nanoshell design configuration that features a dielectric core (ZnO, Si, and SiO_2_) with a radius of 12.5 nm enveloped by a TiN shell with a radius of 25 nm. This proposed design aims to further optimize the efficiency and functionality of solar cells as shown in Fig. 8a. It can be noted that the enhancement in absorption spectra is particularly evident in ZnO specifically at the range from 550 to 650 nm, which can be attributed to its characteristics as a direct band gap semiconductor with an energy gap of approximately 3.4 eV. Additionally, ZnO possesses a substantial exciton binding energy of 60 meV, making it an exceptionally capable semiconductor for a wide range of applications^52,53^. This multipurpose material finds utility in various fields, including UV absorption, antibacterial treatments, solar cells, and photocatalysis. ZnO nanostructures, in particular, have gained significant interest due to their advantageous properties such as low toxicity and economic viability^53^.

To investigate the impact of core radius (core *R*) on absorption peaks, the initial focus of the study is on an isolated single nanoshell within a polymer environment. Figure 8b illustrates the influence of different dielectric core radii, specifically 8 nm, 12.5 nm, 17 nm, and 20 nm. The absorption spectra clearly demonstrate that the highest absorption peak is achieved when the radius of the dielectric core is set to 17 nm. This result suggests that an optimal value for the core radius exists, which maximizes the absorption efficiency of the nanoshell in this particular configuration. The electric field profile of a core–shell NP embedded within a polymer layer is examined at λ = 926 nm. In Fig. 8c, it is evident that the electric field exhibits a concentrated and intense accumulation around the core–shell NP and inside the core. This phenomenon can be attributed to the strong LSPR exhibited by the core–shell NP. The LSPR effect leads to the enhancement and localization of the electric field in the vicinity of the NP, resulting in a highly intense electric field distribution and hence the absorption spectra.

According to Eq. (5), the introduction of PM6Y6 in the OSC structure resulted in an improvement in *J*_*sc*_ compared to the P3HT, as demonstrated in Table 1. Furthermore, the incorporation of TiN NPs within the active layer (PM6Y6) led to a significant enhancement in *J*_*sc*_, approximately 12% higher than that of PM6Y6 alone. Moreover, the utilization of a core–shell nanostructure exhibited an even greater improvement, with *J*_*sc*_ enhanced by approximately 15% compared to PM6Y6 without NPs, as indicated in Table 1.

**Table 1:** The photocurrent density (*J*_*sc*_) and the enhancement percentage for different structures.

Material	*J*_*sc*_ (mA/cm^2^)	Enhancement = $\frac{Jsc(higher) - Jsc(lower)}{Jsc(lower)}\times 100$
P3HT	13.1	–
PM6Y6	27.86	–
PM6Y6 with solid TiN NP	31.14	~ 12% compared with PM6Y6
PM6Y6 with ZnO–TiN core–shell NPs	31.99	~ 15% compared with PM6Y6

**Figure 9:**
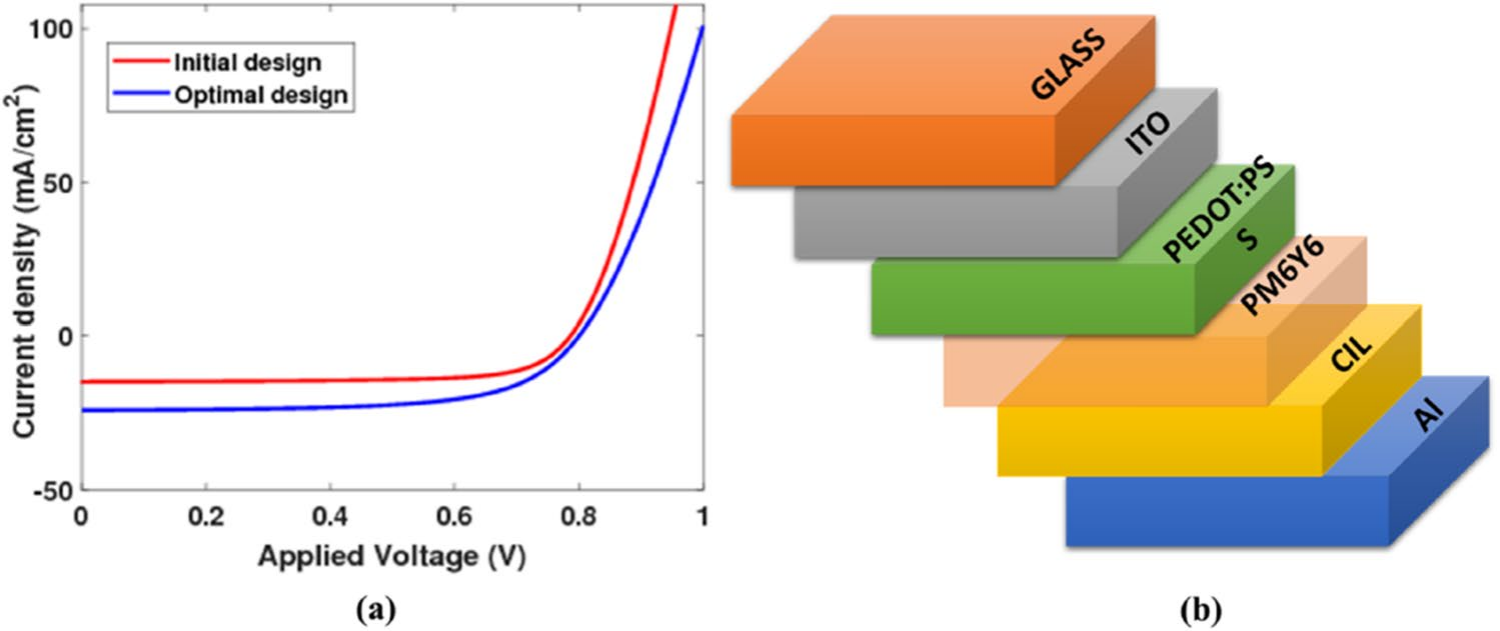
(**a**) *J*–*V* characteristics of planar OSCs and (**b**) 3D structure of the proposed OSC with CIL layer.

This research investigates the electrical characteristics of a planar OSC featuring the active layer PM6Y6, as illustrated in Fig. 1, through the utilization of the OgmaNano software^54^. The study involves an in-depth analysis of OSCs with varying thicknesses of the OSC layers. Through simulation, the investigation aims to assess and graphically represent the relationship between current density–voltage (*J*–*V*) curves, as well as to explore the power conversion efficiency (PCE) of the solar cell by examining parameters such as open circuit voltage (*V*_*oc*_), *J*_*sc*_, and fill factor (*FF*). The simulation process incorporates input parameters with structure ITO/PEDOT:PSS/PM6:Y6/Al derived from the materials used in the OSC, as detailed in reference^55^. The initial thicknesses of the layers as outlined in Table 2 have resulted in the generation of the current density–voltage (*J*–*V*) characteristic curve depicted in Fig. 9a. This curve showcases a PCE of 8.38%, an *FF* of 71.7%, a *V*_*oc*_ of 0.7865 V, and a *J*_*sc*_ of − 14.851 mA/cm^2^. The optimization technique employed in our simulation involves maintaining the thicknesses of the layers while systematically adjusting one parameter at a time until achieving the optimal combination that yields maximum PCE and the desired *J*-*V* curve, as illustrated in Fig. 9a. Due to the square resistance of the ITO layer increasing as the thickness decreases, the ITO layer thickness was kept at 180 nm in line with the initial design^56^.

**Table 2:** Electrical performance characteristics of planar OSCs for the initial thicknesses and the optimal design extracted from OgmaNano^54^.

Case	PM6Y6	PEDOT	ITO	Al	CIL	*V*_*oc*_ (V)	*J*_*sc*_ (mA/cm^2^)	*FF %*	PCE %
Initial design	250 nm	50 nm	180 nm	180 nm	Not found	0.786	-14.85	71.7	8.38
Optimal design	105 nm	20 nm	180 nm	50 nm	10 nm	0.8	-24.15	64.28	12.43

The cathode interfacial layer (CIL) is a crucial component in improving the efficiency and lifetime of BHJ polymer solar cells^57,58^. The thickness of the cathode layer plays a significant role in altering key cell parameters such as *V*_*oc*_ and *J*_*sc*_. In this study, ZnO was chosen as the material for the cathode interfacial layer due to its favorable properties as shown in Fig. 9b^57^. Through this iterative process, we were able to attain a PCE value of 12.43%, an *FF* of 64.28%, *V*_*oc*_ of 0.8 V, and a *J*_*sc*_ of − 24.15 mA/cm^2^. These results highlight the effectiveness of the optimization method in enhancing the performance of the solar cell.

Future studies will involve conducting additional investigations once the experimental electrical characteristics for the PM6Y6, supplemented with core–shell ZnO–TiN nanoparticles, are obtained. According to the literature^42,59^, it is anticipated that the PCE of the OSC incorporating plasmonic NPs will exhibit a potential enhancement. Specifically, the PCE is projected to rise by approximately 1% to 2% compared to the efficiency achieved by the OSC without NPs.

## Conclusion

We presented a theoretical demonstration showcasing enhanced photocurrent density and broadband absorption in organic solar cells (OSCs) by integrating TiN nanoparticles into NFAs, specifically PM6Y6. Compared to the conventional polymer P3HT:PCBM, the proposed structure utilizing PM6Y6 displayed a broader absorption spectrum, indicating its capability to capture a wider range of light wavelengths crucial for improving solar energy conversion efficiency. Using in-house 3D FDTD software, simulations investigated the influence of nanoparticles (NPs) with varying sizes and shapes. TiN NPs were found to efficiently localize light laterally, enhancing absorption in ultra-thin active layers. By employing TiN NPs with diverse size distributions, multiple plasmon resonances could be excited, further broadening the enhancement. Notably, the OSC incorporating ZnO–TiN core–shell nanoparticles exhibited the most significant absorption enhancement, leading to a 15% increase in short-circuit current. Furthermore, leveraging plasmonic nanostructures to enhance absorption in OSCs holds promise in overcoming their primary limitation of low absorption. The utilization of cost-effective and readily available materials like TiN could significantly streamline the commercialization of these devices.

## Data Availability

The datasets used and/or analyzed during the current study are available from the corresponding author upon reasonable request.
